# Conservation of Regional Gene Expression in Mouse and Human Brain

**DOI:** 10.1371/journal.pgen.0030059

**Published:** 2007-04-20

**Authors:** Andrew D Strand, Aaron K Aragaki, Zachary C Baquet, Angela Hodges, Philip Cunningham, Peter Holmans, Kevin R Jones, Lesley Jones, Charles Kooperberg, James M Olson

**Affiliations:** 1 Division of Clinical Research, Fred Hutchinson Cancer Research Center, Seattle, Washington, United States of America; 2 Division of Public Health Sciences, Fred Hutchinson Cancer Research Center, Seattle, Washington, United States of America; 3 Department of Molecular, Cellular and Developmental Biology, University of Colorado at Boulder, Boulder, Colorado, United States of America; 4 Medical Research Council Centre for Neurodegeneration Research, Department of Psychological Medicine, Institute of Psychiatry, King's College London, London, United Kingdom; 5 Life and Bio-Medical Sciences, King's College London, United Kingdom; 6 Department of Psychological Medicine, Wales School of Medicine, Cardiff University, Cardiff, Wales, United Kingdom; 7 Institute of Medical Genetics, Wales School of Medicine, Cardiff University, Cardiff, Wales, United Kingdom; University of Oxford, United Kingdom

## Abstract

Many neurodegenerative diseases have a hallmark regional and cellular pathology. Gene expression analysis of healthy tissues may provide clues to the differences that distinguish resistant and sensitive tissues and cell types. Comparative analysis of gene expression in healthy mouse and human brain provides a framework to explore the ability of mice to model diseases of the human brain. It may also aid in understanding brain evolution and the basis for higher order cognitive abilities. Here we compare gene expression profiles of human motor cortex, caudate nucleus, and cerebellum to one another and identify genes that are more highly expressed in one region relative to another. We separately perform identical analysis on corresponding brain regions from mice. Within each species, we find that the different brain regions have distinctly different expression profiles. Contrasting between the two species shows that regionally enriched genes in one species are generally regionally enriched genes in the other species. Thus, even when considering thousands of genes, the expression ratios in two regions from one species are significantly correlated with expression ratios in the other species. Finally, genes whose expression is higher in one area of the brain relative to the other areas, in other words genes with patterned expression, tend to have greater conservation of nucleotide sequence than more widely expressed genes. Together these observations suggest that region-specific genes have been conserved in the mammalian brain at both the sequence and gene expression levels. Given the general similarity between patterns of gene expression in healthy human and mouse brains, we believe it is reasonable to expect a high degree of concordance between microarray phenotypes of human neurodegenerative diseases and their mouse models. Finally, these data on very divergent species provide context for studies in more closely related species that address questions such as the origins of cognitive differences.

## Author Summary

Animal models of human neurodegenerative and psychiatric disorders, particularly mouse models, have assumed a central role in biomedical research aimed at discovering the causes of disease and generating novel, mechanism-based treatments. But to what degree can a mouse brain serve as a model for a human brain? Here we begin to address this question by looking at patterns of gene expression across three corresponding regions of mouse and human brains. We find that within each species, the different regions (motor cortex, striatum, and cerebellum) have very distinct gene expression profiles. It is likely that these differences reflect distinctions in regional neurochemistry and function. We then show that genes that are enriched in one of the three areas relative to the other two in mice have the same pattern of expression in humans. Looking at the relationship between conservation of expression and amino acid sequence, we find that genes showing patterned expression generally have been more conserved than more uniformly expressed genes. This suggests that in the brain, constraints on the evolution of DNA sequence and gene expression can also be particularly high for genes with regional or tissue-specific expression.

## Introduction

Here we compare and contrast gene expression in three different regions of the human brain, motor cortex, caudate, and cerebellum, to identify genes that are differentially expressed between the regions. In other words, we seek to identify genes that show patterned expression. Knowledge of such regionally enriched genes may provide insight into the development and biochemistry of different brain structures. This information may also hold potential biomedical implications. Many neurodegenerative diseases, such as Huntington's disease, have a hallmark regional and cellular pathology affecting one or another of these regions while sparing the others. It is reasonable to assume that unique susceptibilities in disease may relate to distinctive brain gene expression patterns in health.

We also perform a parallel analysis on the functionally and anatomically corresponding regions of the mouse brain, anterior cortex, striatum, and cerebellum. This allows us to begin to compare and contrast patterns of gene expression in these tissues across these two species. Our motivation for this cross-species analysis also has a biomedical consideration. In recent years, mice have become the most important model organism for human neurological and neurodegenerative diseases. The brains of humans and mice are clearly different with respect to size, complexity, and cognitive abilities. The belief that mice can accurately model human neurodegenerative or neurological diseases rests on assumptions about deeper biological similarities between mouse and human brains that have not been systematically examined. While it is impossible to directly compare mouse arrays to human arrays, one may compare patterns of gene expression in several corresponding brain regions. Comparing gene expression patterns is one way to obtain objective and global information on how similar the brains of humans and mice are. A practical use for this comparative cross-species gene expression information is as a baseline for the comparison of microarray phenotypes of human diseases and their mouse models. For example, if expression changes in a human disease and its mouse model have a global correlation of *r* = 0.5, is this the best possible correlation that can be expected or might we reasonably expect more? Obviously, to answer this question it is useful to know what sort of correlation array data from mice and human brains have initially.

Comparative cross-species information of brain gene expression would also seem to have natural implications for studies that address the origin of cognitive differences between humans and other species. It is generally believed that with respect to other species, even our closest relatives the chimpanzees, humans have unique abilities pertaining to language and higher order cognitive functions. Sequencing projects have revealed that the human and chimpanzee genomes are ∼96% identical, that their typical protein amino acid sequences are ∼99% identical, and that both species have essentially the same number of genes [1]. Since an increase in genomic complexity seems inadequate to explain the apparent mental differences between humans and chimpanzees, the idea that these differences may be due to changes in gene expression has attracted new attention [2].

Recent microarray studies have sought to identify general trends in brain gene expression that distinguish humans from other primates [3–10]. The conclusions reached by these studies have been somewhat discordant. One study found more expression differences between human and chimpanzee liver than prefrontal cortex, but by using orangutan liver and cortex as out groups, concluded there had been an accelerated rate of change in brain gene expression during human evolution [3]. A reanalysis of this data supported this interpretation of rapid and recent human evolution [6]. Other investigations have found that elevated levels of gene expression further distinguish the human brain from that of other primates [4–6]. Several studies have cast doubt upon these findings, attributing them to improper array normalization or to hybridization artifacts rooted in measuring nonhuman primate expression with arrays designed for human sequences [7–10].

Since we are comparing human and mouse expression, we cannot make definitive statements regarding differential expression between humans and chimpanzees. However, examination of global similarity of expression patterns in species as divergent as mice and humans can provide useful context for studies that aim to correlate expression changes with cognitive differences between more closely related species. Presumably, if gene expression patterns distinguish human brains from the brains of other primates, then changes due to recent human evolution may be even more apparent when comparing humans to mice.

## Results

### Absolute and Relative Gene Expression Levels in Equivalent Human and Mouse Brain Structures

The first part of our analysis focuses on expression in different regions of nondiseased brain. We determined absolute and relative gene expression in three anatomically distinct regions of human brain: motor cortex (Brodmann area 4, BA4), caudate nucleus, and cerebellum. The data consisted of samples of all three tissues from 12 donors. These samples constituted a portion of the control group in a study comparing gene expression in Huntington's disease and non-Huntington's brain [11]. For the present reanalysis, the 36 arrays were normalized using Robust Multiple-array Average (RMA) [12]. To assess differential expression between brain regions, three sets of paired t-tests were performed; caudate-to-cerebellum, BA4-to-cerebellum, and BA4-to-caudate using the Bioconductor package LIMMA (http://www.bioconductor.org/packages/1.9/bioc/html/limma.html) [13,14]. To confirm the primary human data, we used a second set of caudate and cerebellum samples from nine different donors [11]. Because of the original study's design, there were no motor cortex samples from these nine donors. The absolute and differential expression analysis of the human samples is provided in Datasets S1 and S2. A key that associates samples with GEO accession numbers, age, gender, post mortem delay, and other covariables can be found in Table S1.

Comparing the log_2_(fold change) (i.e., log ratio) for the replicate caudate-to-cerebellum comparisons indicated that these independent data were highly correlated, with Pearson's correlation coefficient *r* = 0.93 (Figure S1). In the primary caudate-to-cerebellum comparison, 9,088 probesets met *p* < 0.001 with respect to differential expression. In the smaller secondary dataset, 8,074 probesets met *p* < 0.001. Of these, 82% (6,589/8,074) met *p* < 0.001 in both comparisons, and only four probesets showed discordant directions of change. These results demonstrate that the caudate and cerebellum have quite distinct gene expression profiles. They also show that the relative differences between the regions were robust and reproducible in these post mortem human samples. This is consistent with results from a detailed analysis of the relationship between prehybridization variables and posthybridization assessments of data quality, which found little negative contribution from post mortem interval to data quality in these samples [15].

We next performed identical comparative analyses of anterior cortex, striatum, and cerebellum samples from six five-week old wild type C57BL/6 mice. It is generally accepted that these mouse brain regions are anatomically and functionally homologous to human motor cortex, caudate, and cerebellum respectively. We used young mice since very often identifying the earliest changes in a mouse neurological disease model is of primary experimental interest. The complete mouse RMA and regional comparison data are provided in Datasets S3 and S4. Counts of probesets meeting *p* < 0.001 for differential expression in one region relative to the others for both the mouse and primary human data are shown in Table 1. As was found with the human analysis, the three mouse brain regions examined had very distinct gene expression profiles with many statistically significant changes (Table 1).

**Table 1:**
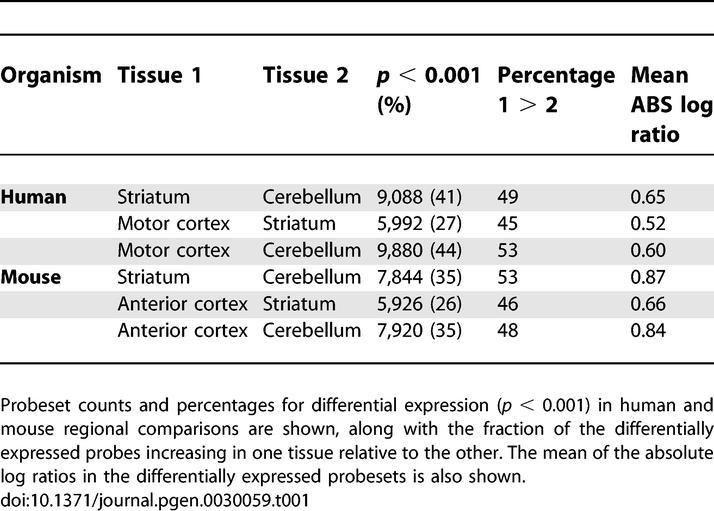
Different Regions of the Brain Show Many Statistically Significant Differentially Expressed Genes

To provide additional verification of the data, we queried the human and mouse caudate/striatum-to-cortex and caudate/striatum-to-cerebellum comparisons against a published list of 54 striatum-enriched mouse genes (Table S2) [16]. Table 2 shows both the human and mouse array data to be consistent with known mouse striatal genes (*p* ≈ 10).

**Table 2:**
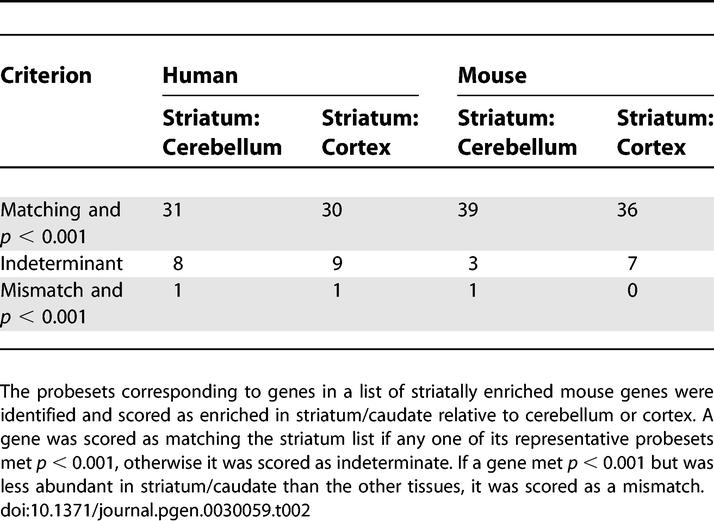
Mouse and Human Microarray Data Are Consistent with Previously Identified Striatal Genes

Table 3 shows the 30 named genes with the highest regional scores (see Materials and Methods) in each species. At their most extreme, the pattern of expression for these genes is “on” in one region and “off” in the other two regions. Table 3 represents only a small subset of regionally enriched genes, and the complete data pertaining to differential expression between brain regions can be found in Datasets S2 and S4. Several genes appear in both the mouse and human lists. Even considering these short lists of top regional genes, the intersections between human and mouse gene lists are highly statistically significant (*p* < 10^−7^ for each intersection).

**Table 3:**
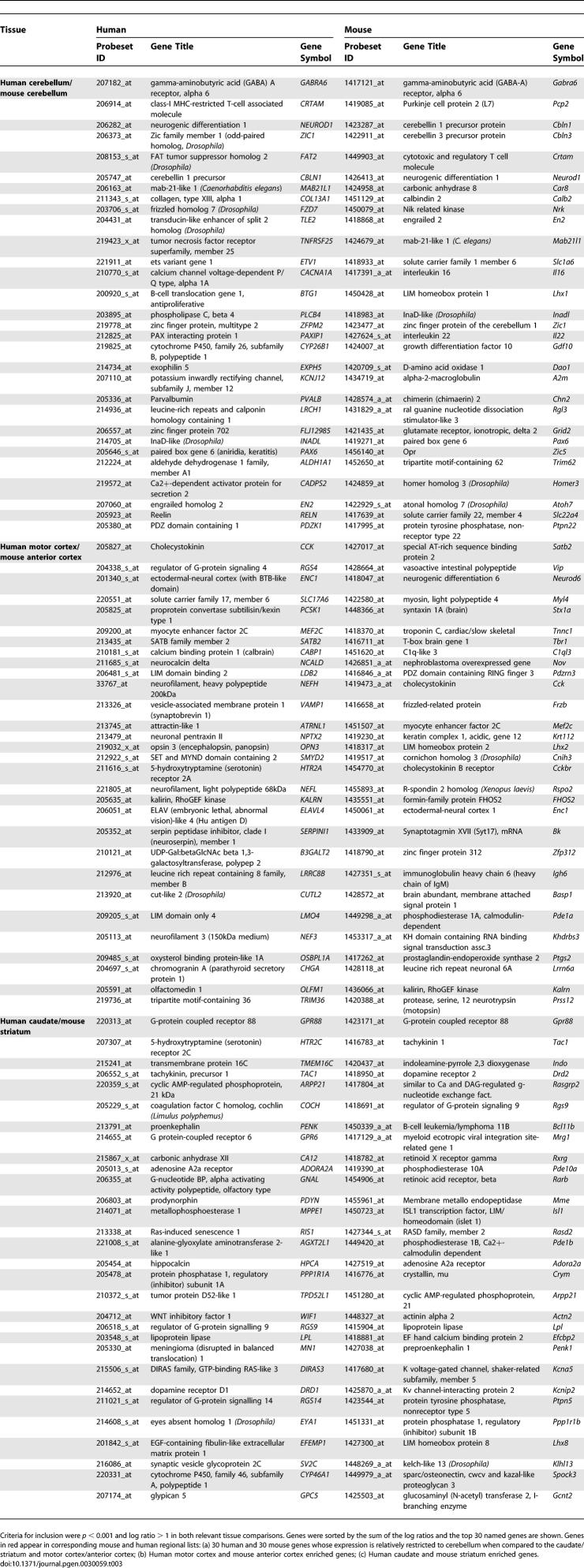
Selected Regionally Enriched Genes in Human and Mouse Brain Tissues

### Gene Expression Variation between Tissues and Individuals

While the primary interest of this study was in regional differences, many factors such as age, gender, tissue heterogeneity, post mortem interval, medication, and cause of death may influence gene expression in the brain and contribute to differences between individuals. To examine the effect of individual variability of the gene expression on the profiles, the between-tissue and within-tissue variances for each probeset were computed from the human RMA signals. This was repeated for the mouse probesets. As post mortem delay was not a concern with the mouse samples, and all of the mice were the same age, mouse individual variability might reasonably be expected to be smaller than human variability. We found that the between-tissue variability was greater for 89% of the human probesets and 85% of the mouse probesets. This suggested that human individual variability in gene expression and factors such as tissue heterogeneity and post mortem interval were not obscuring or significantly contributing to regional differences. It also suggested that compared to expression dictated by regional identity, age and gender appear to have effects of small magnitude or of large magnitude on a small fraction of genes, even in humans. Some evidence for this can also be inferred from the two independent human caudate-to-cerebellum comparisons. In these comparisons, age and gender were not balanced between the groups, yet relative expression levels were highly correlated and the slope of the regression line was 0.967 (Figure S1).

### Cross-Species Comparison of Regional Gene Expression

To explore the gene expression of each tissue in more depth, we used gene ontology (GO) [17]. GO provides means of objectively identifying functional themes in large groups of genes, in this case the genes that were differentially expressed in the pair-wise regional comparisons within each species. A significantly high number of overrepresented GO categories were found for both human and mouse in all three types of regional comparisons. This was true whether considering increased or decreased probesets separately or together.

While GO is not intended for rigorous assessment of evolutionary relationships, GO nomenclature is standardized. This allowed us to compare and contrast the regionally enriched functions in the homologous mouse and human brain regions. Of the hundreds of GO categories differentially represented in one region relative to another, many were common between the corresponding human and mouse comparisons (Tables 4 and S3). Permutation testing showed these intersections to be significantly greater than would be expected by chance (*p* < 0.0001) (Table S4).

**Table 4:**
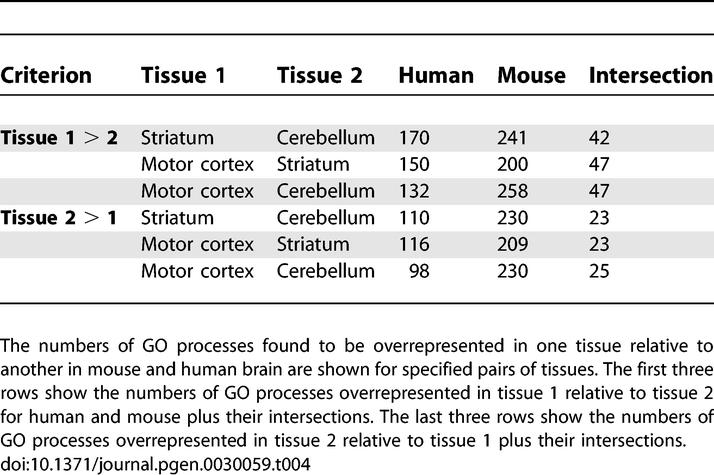
Mouse and Human Brain Regions Share a Higher Number of Overrepresented Functional Groups than Would Be Expected by Chance

Both the functional GO analysis and the intersections among top regional marker genes hinted that relative gene expression levels across brain regions have been conserved between mice and humans. To examine this in depth, it was necessary to contrast expression ratios on a gene-by-gene basis across the human and mouse arrays. This was complicated by the fact that genes are often represented by more than one probeset on each array. To lessen this complication for our initial analyses, genes were identified where only one probeset existed on each array. We also arbitrarily required that human and mouse gene symbols were identical, since it was a clean and simple way to identify genes that have met widely accepted criteria for being orthologous pairs. Using these criteria, 2,998 one-to-one orthologous pairs were found on the mouse and human arrays.

**Figure 1:**
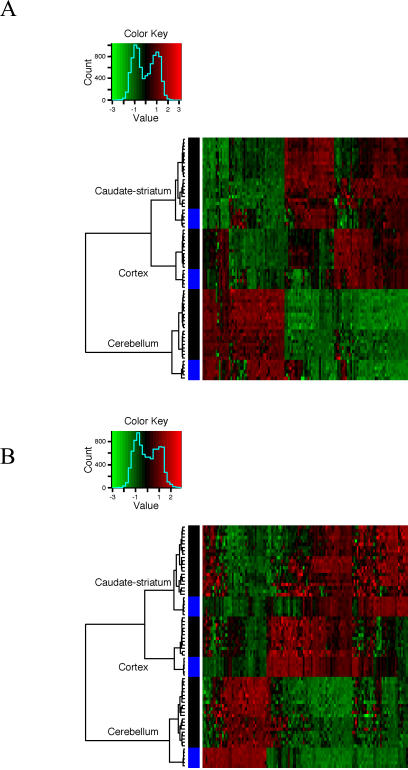
The Most Variable Genes in One Species Accurately Cluster the Brain Regions of the Other Species, and Orthologous Structures Cluster Together (A) Hierarchical clustering of regions and species using the 125 human genes in the one-to-one set with the highest variance across the 36 primary human arrays is shown. (B) Hierarchical clustering of regions and species using the 125 mouse genes in the one-to-one set with the highest variance across the 18 mouse arrays is shown. While variable genes were identified using only the primary human data, clustering included the secondary set of human data. The mouse, primary, and secondary human data were normalized separately. Expression levels are colored by standard deviation from the within-group mean. Columns represent genes. Rows represent samples with black bars on the left signifying human samples, and blue signifying mouse samples.

Taking this set of genes, we first asked whether genes with high variance of expression across the brain regions of one species would cluster the entire set of samples sensibly. This was motivated by our earlier observation that the largest component of a gene's expression variance was due to tissue specificity. Thus, high variance implied patterned expression across the three brain regions. Figure 1 shows that both the mouse and human genes with the largest variance in the one-to-one gene set cluster all of the samples perfectly, first by tissue, then by species. In other words, for these three brain regions, the equivalent human and mouse regions are more alike than different regions within a species or individual. We also note that while we selected the genes based on variability of expression across regions within a species and not conservation between species, there were 43 genes in common on the two lists of 125 most variable one-to-one genes (*p* ≈ 0). The heat maps of normalized expression indicated that relatively few genes in corresponding brain regions were on opposite sides of their mean signal within a species. All of these observations suggest a high degree of similarity in the genes with patterned expression in mouse and human brain.

Using all genes in the one-to-one set, we next examined relatedness of regional gene expression within and between species by computing normalized Euclidian distances between all possible nonself pairs of tissues (Table 5). The similarity of corresponding tissues between the species was apparent by their consistently having the minimal between-species distance. The pattern of distances between regions within the human brain was essentially identical to the pattern of distances within the mouse brain, suggesting that no single region of the human brain had diverged from the other two regions any more than regions in the mouse brain had diverged from each other.

**Table 5:**
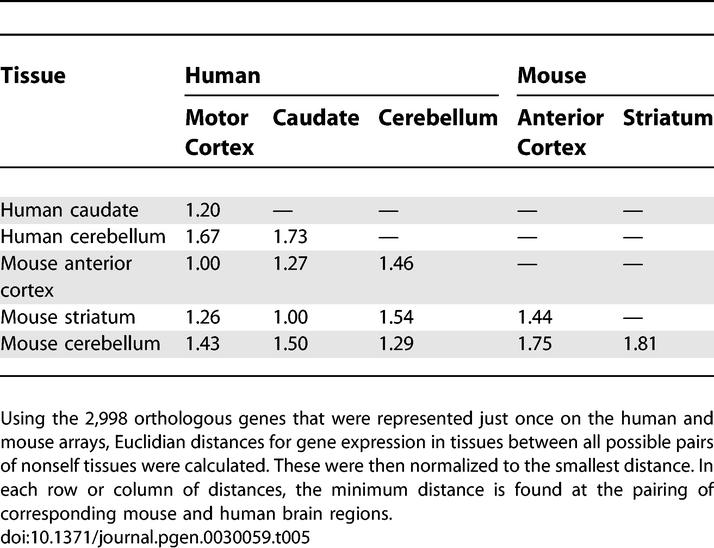
Orthologous Brain Regions between Species Are More Similar to Each Other than to Different Regions within a Species

To expand our analysis beyond the one-to-one subset of genes, ENSEMBL (http://www.ensembl.org) information was used to identify a more complete set of mouse-human orthologs. Where more than one probeset represented a gene, we retained only information pertaining to the probeset with the highest mean RMA signal. This collapsed the arrays to a nonredundant set of 8,499 genes common to both array types (Dataset S5). We then correlated log ratios in the appropriate pairs of tissue comparisons over all the genes. The correlation coefficient of the mouse and human caudate-to-cerebellum log ratio was *r* = 0.47. For the cortex-to-cerebellum comparisons and for the cortex-to-caudate comparisons, *r* = 0.45.

### Relationships between Tissue-Specific Expression, Conservation of Sequence, and Conservation of Expression

**Figure 2:**
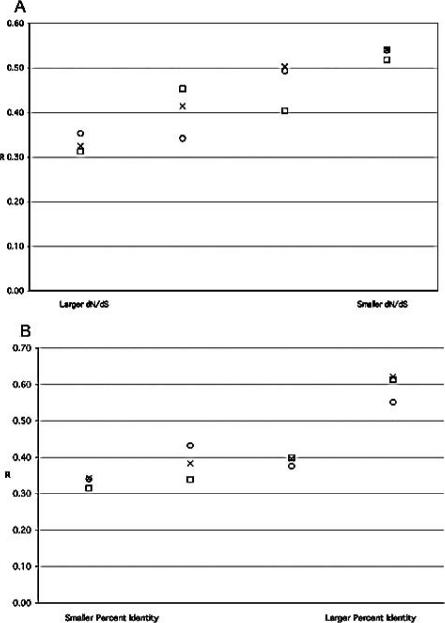
Conservation of Gene Expression Increases with Conservation of Sequence (A) Othologous human and mouse genes were ranked by their dN/dS ratios, as given in the ENSEMBL database, from least conserved at the DNA sequence level (larger dN/dS), to most conserved. For each of the three pair-wise regional comparisons, the correlation coefficient between human and mouse log ratios was determined and plotted for each quartile of genes. (B) Orthologous genes were ranked by their percent nucleotide identity, as given in the ENSEMBL database, and quartile correlation coefficients were determined and plotted for each quartile of genes. X, caudate or striatum-to-cerebellum; Circle, cortex-to-cerebellum; Square, cortex-to-caudate or striatum.

We explored the hypothesis that genes whose sequences had been under stabilizing selective pressure would also be constrained in their pattern of expression. Information about nonsynonymous and synonymous amino acid substitution ratios (dN/dS) and percent nucleotide identity for mouse and human orthologs were retrieved from the ENSEMBL database. The set of 8,499 orthologous genes was ranked by each of these metrics, and a correlation coefficient between appropriate human and mouse log ratios was computed for each quartile of genes. The quartile-based correlation coefficients are plotted for each class of tissue comparison in Figure 2. This shows that there is a positive relationship between conservation of sequence and conservation of expression.

**Figure 3:**
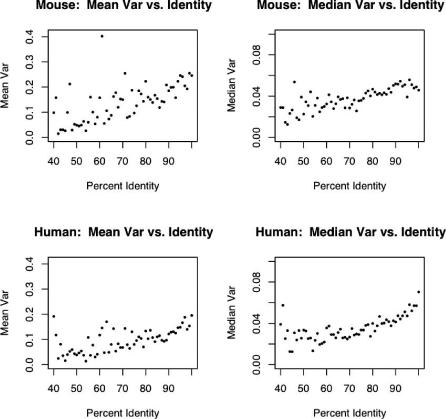
Genes with High Variance across Tissues Have Greater Conservation of Nucleotide Sequence Orthologous mouse and human gene pairs were binned according to their nearest integral percent identity. The mouse or human mean and median variance of each bin are plotted against the bin's percent identity.

It seems natural to assign greater confidence in a pairing between two genes that are 95% conserved at the sequence level than between two genes that are 75% conserved. Furthermore, as homology thresholds decrease, the number of potential ortholog pairings increases. Because of these factors, we assume that our rate of incorrectly pairing orthologs may increase as percent nucleotide identity decreases. Pairing errors also likely reduce correlation, thus any bias in the error rate of pairing may introduce a false positive relationship between homology and gene expression. To avoid this potential bias, we examined the relationship between variability of expression across tissues and sequence conservation. Results presented above suggest that in mouse and human brain, the genes with the greatest variability of expression in the three examined brain regions were similar (Figure 1). We also showed that expression variance was most strongly dependent upon tissue specificity rather than variability between individuals. Variance within a species can be determined in the absence of homology information, so we examined the within-species variance of bins of genes with integral percent identities. Figure 3 shows that there is a clear tendency in both species for genes with higher expression variance across brain regions to have higher identity with their orthologs. Since expression variance is a surrogate for tissue specificity, this indicates that region-specific genes in the brain tend to have greater homology with their orthologs than more widely expressed brain genes. This is consistent with the idea that functional constraints have applied selective pressure on brain gene expression since the mouse and human lineages diverged some 80 million years ago.

## Discussion

Our data indicate that expression patterns across comparable regions of human and mouse brains have generally been conserved since the two lineages diverged. This is consistent with classical comparative neuroanatomy, which has long indicated general conservation of gross mammalian brain structure and conservation of cell types within comparable regions [18,19]. Conservation of patterned gene expression in the mammalian brain is consistent with standard assumptions of biological uniformity justifying the use of model organisms. Further underscoring conservation of mammalian brain gene expression, we find that in the three brain regions examined, equivalent regions in mouse and human brain are more alike than different regions within a species. This is apparent whether considering the 125 genes with the most variable expression within a species (Figure 1) or whether considering Euclidian distances based on expression of thousands of orthologous gene pairs (Table 5). Our finding is consistent with other studies contrasting brain gene expression in dogs and humans [20], chimpanzees and humans [7], and mice and humans [10].

We do not mean to suggest, and our findings should not be interpreted to mean that gene expression in human and mouse brains is identical. Here we are mainly concerned with the genes that show an extremely patterned expression across three particular brain regions. Because our study examines expression of the tissue, we cannot discern evolutionary changes within specific cell types. However, within the three regions examined here, the overall trend is for regional-marker genes to have been conserved. For example, considering the human motor cortex-to-cerebellum comparison, in the 100 human genes with greatest evidence for differential expression between these regions, there are only nine discordant changes in the 100 mouse orthologs. The overall correlation between human and mouse log ratios in this set of 100 genes is *r* = 0.86. In the top 250 human caudate-to-cerebellum changes, *r* = 0.81, and there are 39 discordant changes. In the top 500 changes, *r* = 0.75, and there are 93 discordant changes. Essentially identical correlations and trends appear in cross-species correlation of the other two regional comparisons (unpublished data). It is very likely that our data somewhat underestimate the true correlation, since factors such as post mortem delay, tissue dissection, and gender ratios were not strictly controlled. Other technical sources of variability include possibly measuring different splice variants in mice and humans, comparing young mice to old humans, and differences in cell-type composition arising from comparing whole mouse tissues to small portions of the human tissues. Finally due to evolution of genomic sequence, Affymetrix (http://www.affymetrix.com) must almost always use probes of different sequences to assay human and mouse gene expression. Probe sequence has a profound influence upon the signal detected in a microarray experiment [21].

Overall we observe correlation of relative expression levels in mice and humans on the order of *r* = 0.45. This leaves the proportion of unexplained variance due to technical factors and evolutionary changes as roughly 80% (1 − 0.45^2^ = 0.8). If an estimate of the variance due to technical factors can be made, in theory it is possible to determine the proportion of unexplained variance due to the evolution of gene expression. From the correlation observed in our independent human regional comparisons, one can arrive at an estimate of 14% of the variance being due to technical noise for a within-species regional comparison (1 − 0.93^2^ = 0.14). Perhaps twice this or 28% may serve as an estimate of cross-species technical noise. Thus at the high end, our data suggest 52% (80%−28%) of the variance could be due to evolutionary changes. It may be more accurate to suppose that cross-species correlations are subject to the same technical noise effects as within-species correlations on different generations of Affymetrix microarrays. In that case, typically *r* = 0.7 [21]. Therefore, the estimate of variance due to noise is 1 − 0.7^2^ = 0.51, which leaves 0.8 − 0.51 = 0.29 or 29% as our estimate of the unexplained variance due to evolution of expression in mice and humans. Since we are examining log ratios, evolutionary contributions from both tissues in each species are combined in this number. We presume the true variance due to evolution within each single tissue would be less than 29%.

It might be reasonable to expect that gene expression variability would be significantly larger between individual humans than between inbred mice housed in uniform conditions. There is little evidence for this in our profiles. We find the fraction of genes that vary between individuals more than between tissues is roughly the same in the two species. These findings could be unique to the regions examined, or they may be a consequence of the between-region variability being so much larger than individual variability for both humans and inbred mice. A more interesting alternative is that this implies that the constraints on brain gene expression are quite strict and that many commonly presumed sources of individual variability are just not that influential. Gender may be one of the largest contributors to individual gene expression variability. In analyses to identify gender-dependent gene expression differences in human brains, differential expression was limited to a rather small set of genes when the X and Y chromosomes were excluded (L. Jones, unpublished data).

Examining orthologous mouse and human genes, we find that conservation of amino acid and nucleotide sequence is correlated with conservation of regional expression. Since this relationship could have been an artifact of our ability to identify homologous genes, we re-examined this relationship by beginning with genes that showed evidence for regional expression within one or the other species. This showed that the genes with higher variability of expression between brain regions within a species also tended to have greater sequence homology with their orthologs than genes that are expressed in multiple brain regions. This is somewhat surprising if one imagines that evolutionary constraints act additively on genes widely expressed in different tissues. It may be that regional gene expression in the brain is particularly highly constrained since the proper behavior of the organism depends upon each brain region functioning smoothly with the others. Wider surveys of tissue gene expression tend to support constraints on brain gene expression, finding that the brains of humans and chimpanzees show fewer differentially expressed genes than kidney, heart, liver, and testes [3,22].

Particular interest has been devoted to finding differences between humans and chimpanzees. Some studies have concluded that there is a bias for genes to be more highly expressed in human cortex relative to chimpanzee [4,6]. While our data cannot directly address chimpanzee and human gene expression, and this claim was made for a rather small number of genes, we see little evidence that the human cortex has uniquely undergone extensive and rapid evolutionary change. Based on the Euclidian distances shown in Table 5, we find it is the cerebellum that is the outlier tissue both within and between the two species.

It is quite possible that complexity of higher order brain functions relate to splicing or protein modifications that escape microarray analysis, but our data suggest some boundaries on the idea that gene expression differences explain differences in cognitive abilities between species. Few genes appear to have evolved new patterns of regional expression. The minority of genes that do show discordant regional expression between adult mice and humans may indeed be key genes regulating brain functions. Alternatively, since general expression patterns in the adult brain have largely been conserved, perhaps it is gene expression during development that ultimately wields the most influence upon higher brain functions by specifying the complexity of neuroanatomy. Humans have at least two orders of magnitude greater numbers of neurons and neuronal connections than mice [18,19]. Our data suggest the active genes in those neurons and connections are quite similar in adult mice and humans, species with extremely different cognitive abilities. This similarity should become greater as more closely related species, such as chimpanzees and human, are considered. The most important genes relating to cognitive differences may be genes that specify how the machinery is assembled.

Transgenic mice have become the most common model organism for human neurodegenerative diseases [23]. Scrutiny of models has previously involved comparing histopathological and neurochemical phenotypes, or extrapolating from mouse neurobehavioral tests to human disease signs and symptoms. We suggest that the transcriptional signature of the human disease can be used to objectively and globally assess both genetic and phenotypic models; the assumption being that a model that recapitulates the human disorder should have a similar expression profile. Ideally, such assessment involves reference to a range of expression profiles so that the biological specificity of the disease phenotype can be addressed and to provide outlier groups to place relatedness in context [24]. We believe that contrasting healthy mouse- and human-brain gene expression profiles provides a reasonable context with which to assess likeness between mouse models and human neurodegenerative diseases. The high correlation between regional gene expression in healthy brain suggests that mouse models of human neurodegenerative diseases may quite accurately recapitulate the human microarray phenotype and should be held to a high standard.

Here we have focused on the general similarity rather than specific differences between two species. Using several different methods, we find that regional gene expression in the mouse anterior cortex, striatum, and cerebellum is very similar, respectively, to gene expression in human motor cortex, caudate, and cerebellum. Classical comparative neuroanatomy has identified a general conservation of mammalian brain structure, with differences between species arising from elaboration of ancestral forms. Our data indicate that this general conservation continues down to the gene expression level, and that expression patterns in our brains may be less far removed from ancestral forms than apparent differences in mental abilities might suggest.

## Materials and Methods

### Human tissue dissection and RNA processing.

Post mortem human tissue was gathered with ethical approval and permissions, dissected, and processed as specified [11]. The samples were hybridized to Affymetrix HG-U133A arrays containing 22,283 probesets. The primary dataset consisted of caudate, cerebellum, and motor cortex samples from eight men and four women, whose ages ranged from 36 to 77 with an average age of 58 years. Confirmation of the primary data was performed with an independent second group that consisted of caudate and cerebellum samples from seven men and two women whose ages ranged from 22 to 72 with an average of 49 years. Clustering included all human and mouse samples.

### Mouse tissue dissection and RNA processing.

Postnatal day-35 C57BL/6 mice, five females and one male, were killed by cervical dislocation. The brain was immediately dissected into ice-cold phosphate-buffered saline. Tissue microdissections were performed at 4 °C on one hemisphere at a time with the brain on a bed of dry ice. The cortex was divided into an anterior and posterior portion with the line of division at the point where the striatum and hippocampus meet. Tissue was collected into 5-ml polypropylene Falcon tubes, submerged in liquid nitrogen, and stored at −80 °C. Total RNA was isolated by adding 1 ml of Qiazol reagent (Qiagen, http://www.qiagen.com) to each frozen sample and homogenizing the tissue with a polytron for 40 s at medium speed. Residual salts and proteins were removed with an RNeasy Lipid Kit per the manufacturer's instructions (Qiagen). RNA concentration was determined with spectrometer. The Affymetrix single-cycle probe synthesis kit was used to generate cRNA probe per the manufacturer's instructions. For the cortical and cerebellar samples, 5 μg of total RNA was used as starting material. For striatal samples, 2 μg of total RNA was used. Biotinylated-cRNA was checked on a bioanalyzer prior to and after the fragmentation reaction. Samples representing tissue from a single mouse were hybridized to MOE_430A_2 chips containing 22,690 probesets, *n* = 6 for each tissue. The raw image data is available at http://www.hdbase.org.

### Microarray analysis.

Primary analysis of microarray data was performed using Bioconductor, a freely available software package designed for the analysis of genomic data (http://www.bioconductor.org). We first preprocessed and normalized the CEL files with RMA*.* The primary and secondary groups of human samples were normalized and analyzed separately. Then we fit a linear model (gene expression ≈ donor + tissue type) for each of the three paired comparisons of tissue using the Bioconductor library package LIMMA to calculate log ratios, moderated paired *t*-statistics, and corresponding *p*-values. We did not further adjust *p*-values for multiple testing. Here we primarily used *p*-values for ordering genes. Additional adjustments, such as a Bonferroni or Benjamini-Hochberg correction, would not affect how we ordered genes since such adjustments are typically monotonic operations.

To select sets of genes whose expression was highly enriched in one of the three regions under consideration, we chose as arbitrary criteria that probesets met *p* < 0.001 and log ratio ≥ 1 in both relevant pair-wise comparisons. To rank probesets, the log ratios of the two relevant comparisons were summed in the appropriate fashion to provide a positive regional score. For example, the largest values of log_2_(BA4/caudate) + log_2_(BA4/cerebellum) would be candidate BA4 genes. Finally, probesets whose summed regional score was >2 in more than one region were culled from the list.

The variance for a probeset, across *n* samples, was calculated by

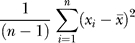
where *x*_i_ is the RMA signal for probeset *i* on array *n*. After selecting variable genes, to minimize systematic differences of scale between the mouse and human arrays, prior to clustering we separately normalized the mouse and human RMA data to give each probeset zero mean and unit variance. Hierarchical clustering and heat maps using the 125 (an arbitrary number) most variable probesets were generated using Ward's linkage method, which uses an analysis of variance approach to evaluate the distances between clusters. In short, this method attempts to minimize the sum of squares of any two (hypothetical) clusters that can be formed at each step [25]. Heat maps were generated with the Comprehensive R Archive Network (CRAN) package GREGMISC.

Euclidean distances between samples were calculated using RMA signals by

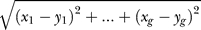
where there are g probesets and *x* and *y* are any two mouse or human samples. Euclidian distances between regions were calculated using the mean RMA probeset signals for each tissue.

### Bioinformatics.

To extract ortholog identities, the ENSEMBL database (http://www.ensembl.org/Multi/martview) was queried using mouse ENSEMBL identities provided in the Affymetrix annotation. Human ENSEMBL numbers, dN (number of nonsynonymous substitutions/number of nonsynonymous sites), dS (number of synonymous substitutions/number of synonymous sites), dN/dS, and percent identity were retrieved and associated with mouse probesets. dN and dS values were generated using the codeml program included in the PAML package [26,27]. Codeml performs pair-wise Maximum Likelihood calculations of dN and dS for each set of orthologs. We used the F3 × 4 codon evolution model. This takes into account bias derived from the different probabilities of transition versus transversion mutations and bias due to different nucleotide frequencies at the three codon positions. Incorrect ortholog assignments manifest as anomalously high dS values. We therefore applied a cut off of twice the median dS as the criterion for retaining the dN/dS ratio.

### Statistical methods.

Since a large but unknown fraction of genes are coregulated, assumptions of independence are not met. We therefore report extreme statistical significance (*p* < 10^−20^) as *p* ≈ 0, as we do not wish to imply that we believe all assumptions are correct. While additional computation might improve our estimate of *p,* results when assuming independence are so extreme that our conclusions per statistical significance would not change.

*p*-Values for the intersections of lists of regional marker genes in Table 3 were calculated assuming a hypergeometric distribution drawing two lists of 30 from a pool of 8,500 genes. *p*-Values for intersection of most variable mouse and human genes in Figure 1 were calculated assuming a hypergeometric distribution drawing two lists of 125 genes from a pool of 2,998 genes. *p*-Values for correlation coefficients were calculated with a likelihood ratio test assuming observations are independent realizations from a joint bivariate normal distribution.

### Analysis of GO categories in different regions.

Categories overrepresented in lists of probes were differentially expressed between different tissue regions (e.g., caudate versus cortex) within species. For the human HG-U133A arrays, 70.6% of the probesets had an assigned GO category. For the mouse MOE430A_2 arrays, 66.2% of the probesets had an assigned GO category. For each GO category, the total number of probes in that category and the number of probes appearing on a list of differentially expressed probes (*p* < 0.05) were calculated. A *p-*value for overrepresentation of each category was calculated using Fisher's exact test if either the number of probes on the list or the number not on the list was less than ten, otherwise a Pearson chi-square was used. The number of categories achieving a given *p-*value for overrepresentation was calculated, and its significance assessed by permutation (to account for the overlap in categories). The permutation procedure was as follows: generate a list of differentially expressed probes of equal length to the actual list by sampling probes at random (without replacement); calculate the number of probes on the list for each GO category, and hence a *p-*value for overrepresentation; count the number of categories with a *p-*value for overrepresentation less than the specified criterion, and compare to that in the actual data; repeat the process 5,000 times.

Overlaps in overrepresented categories between species for a given regional comparison were examined. These analyses were restricted to the 3,119 GO categories defined for both human and mouse. The number of categories significantly overrepresented (*p* < 0.05) for both mouse and human in the actual data was calculated for each comparison and direction of expression. Significance was again assessed by permutation (to reflect the fact that several probes are in more than one category). A random list of differentially expressed probes of equal length to that observed in human was generated and used to calculate *p-*values for overrepresentation for the human GO categories, as before. The *n* most significant categories were selected (*n* being the number of significantly overrepresented categories in the actual human data), and the overlap between these and the significantly overrepresented mouse categories calculated. The process was repeated 10,000 times. For all three regional comparisons and all expression directions, the number of overlapping categories in the actual data was higher than that obtained in any of the simulated replicates.

## Supporting Information

Dataset S1 Complete Data for Human RMA Signals (8.8 MB ZIP)

Dataset S2 Complete Data for Human Regional Comparisons (6.0 MB ZIP)

Dataset S3 Complete Data for Mouse RMA Signals (5.1 MB ZIP)

Dataset S4 Complete Data for Mouse Regional Comparisons (5.0 MB ZIP)

Dataset S5 Alignment of Human and Mouse Orthologous Genes Including Differential Expression Statistics from Regional Comparisons (3.8 MB ZIP)

Figure S1 Relative Gene Expression in Human Brain Tissues Is Robust and Reproducible The log ratio from the caudate-to-cerebellum comparison in the primary set of human samples is plotted (x-axis) against the caudate-to-cerebellum log ratio of the independent second set of human samples. The data are highly correlated (*r* = 0.93) despite having unbalanced age and sex ratios between the two groups.
(2.1 MB TIF)

Table S1 Key Relating GEO Accession Numbers, Clinical Covariates, and Samples (31 KB XLS)

Table S2 Striatal Genes Listed in [16] (34 KB XLS)

Table S3 GO Categories Common to Human and Mouse Brain Regions (59 KB XLS)

Table S4 Observed and Expected Numbers of GO Categories Reaching Various *p*-Value Thresholds and *p* < 0.05 False Discovery Rates (FDR) (14 KB XLS)

### Accession Numbers

The GEO database (http://www.ncbi.nlm.nih.gov/geo) accession number is GSE3790. Affymetrix Web site (http://www.affymetrix.com) annotations for human HG-U133A and mouse MOE430_2 are from (http://www.affymetrix.com/support/technical/byproduct.affx?product=hgu133) and (http://www.affymetrix.com/support/technical/byproduct.affx?product=moe430–20).

## Abbreviations

Brodmann area 4: BA4
dN: number of nonsynonymous substitutions/number of nonsynonymous sites
dS: number of synonymous substitutions/number of synonymous sites
GO: gene ontology
RMA: Robust Multiple-array Average
