# Supplementary material for: Conservation of Regional Gene Expression in Mouse and Human Brain
Source: PLoS Genet. 2007 Apr 20;3(4):e59. doi: 10.1371/journal.pgen.0030059 (PMC1853119; doi:10.1371/journal.pgen.0030059)
Supplement: Supplementary file 1 [file pgen.0030059.st003.pdf]

Significantly over-represented overlapping gene ontology processes in the tissue comparisons in mouse and man. Only categories where the number of probesets in either mouse or human data was >10 are included.

A: human cortex-caudate and mouse cortex-striatum GO processes containing probesets with reduced RNA levels in human and mouse cortex compared with human caudate and mouse striatum respectively

GO ID	Human		Mouse		Function
	# sig/total probesets	p-value	# sig/total probesets	p-value	
6412	141/ 389	0.00E+00	76/ 565	7.34E-14	protein biosynthesis
6414	13/ 29	2.71E-08	6/ 38	2.59E-02	translational elongation
8360	11/ 34	1.32E-04	18/ 118	1.45E-05	regulation of cell shape
30036	22/ 92	2.19E-04	14/ 127	9.66E-03	actin cytoskeleton organization and biogenesis
6869	17/ 77	3.27E-03	15/ 95	3.60E-05	lipid transport
6730	11/ 45	5.15E-03	13/ 69	4.41E-06	one-carbon compound metabolism
6936	24/ 126	7.48E-03	7/ 44	1.60E-02	muscle contraction
8277	11/ 47	8.13E-03	3/ 13	4.04E-02	regulation of G-protein coupled receptor protein signaling pathway
42135	5/ 12	9.54E-03	3/ 8	9.94E-03	neurotransmitter catabolism
187	7/ 22	1.19E-02	9/ 53	4.33E-03	activation of MAPK activity
30154	58/ 369	1.38E-02	55/ 624	1.94E-03	cell differentiation
6629	46/ 286	1.67E-02	36/ 293	3.95E-06	lipid metabolism
6397	17/ 90	2.23E-02	50/ 424	3.62E-07	mRNA processing
6376	6/ 20	2.61E-02	12/ 60	3.09E-06	mRNA splice site selection
6334	14/ 73	2.98E-02	15/ 86	4.96E-06	nucleosome assembly
45786	24/ 144	4.27E-02	13/ 115	9.34E-03	negative regulation of progression through cell cycle
51016	10/ 46	4.37E-02	13/ 63	6.40E-07	barbed-end actin filament capping
910	6/ 23	4.99E-02	5/ 30	3.29E-02	cytokinesis

B: human cortex-caudate and mouse cortex-striatum GO processes containing probesets with increased RNA levels in human and mouse cortex compared with human caudate and mouse striatum respectively

GO ID	Human		Mouse		Function
	# sig/total probesets	p-value	# sig/total probesets	p-value	
6886	94/ 310	5.61E-15	66/ 491	1.24E-08	intracellular protein transport
15986	26/ 53	1.14E-12	16/ 60	1.37E-09	ATP synthesis coupled proton transport
7018	31/ 71	3.86E-12	30/ 140	1.33E-11	microtubule-based movement
7264	70/ 232	1.98E-11	50/ 396	6.39E-06	small GTPase mediated signal transduction
16192	33/ 81	2.51E-11	21/ 140	1.14E-04	vesicle-mediated transport
15992	34/ 87	9.21E-11	23/ 93	1.23E-11	proton transport
51258	18/ 25	1.96E-10	10/ 26	5.49E-06	protein polymerization
6096	26/ 61	4.87E-10	21/ 83	3.79E-11	glycolysis
15031	76/ 300	1.31E-07	103/ 870	1.16E-08	protein transport
6607	16/ 35	1.35E-07	2/ 3	1.42E-02	NLS-bearing substrate import into nucleus
6887	17/ 39	2.14E-07	11/ 81	1.07E-02	exocytosis
6811	91/ 381	2.70E-07	115/ 778	0.00E+00	ion transport
7269	13/ 22	2.19E-06	7/ 47	4.50E-02	neurotransmitter secretion
6813	45/ 173	1.65E-05	58/ 281	0.00E+00	potassium ion transport
8152	87/ 392	1.78E-05	63/ 619	1.06E-03	metabolism
6457	63/ 268	2.94E-05	38/ 397	2.39E-02	protein folding
7254	13/ 33	3.57E-05	6/ 30	1.66E-02	JNK cascade
6413	14/ 37	4.09E-05	11/ 61	3.93E-04	translational initiation
7242	87/ 420	3.12E-04	117/ 834	8.33E-16	intracellular signaling cascade
6470	44/ 188	4.52E-04	39/ 278	2.46E-06	protein amino acid dephosphorylation
6897	29/ 113	5.85E-04	25/ 239	1.91E-02	endocytosis
7268	54/ 244	6.34E-04	16/ 123	4.79E-03	synaptic transmission
46777	9/ 19	7.72E-04	9/ 57	1.75E-02	protein amino acid autophosphorylation
7017	7/ 14	2.06E-03	30/ 142	2.49E-11	microtubule-based process
6754	3/ 3	3.26E-03	18/ 55	4.65E-14	ATP biosynthesis
6816	29/ 126	4.80E-03	25/ 201	1.36E-03	calcium ion transport
16055	18/ 72	7.55E-03	33/ 209	3.61E-07	Wnt receptor signaling pathway
30182	4/ 7	1.16E-02	10/ 49	1.91E-03	neuron differentiation
1558	25/ 116	2.07E-02	20/ 133	1.53E-04	regulation of cell growth
1764	2/ 2	2.20E-02	12/ 98	2.19E-02	neuron migration
7026	5/ 12	2.29E-02	6/ 33	2.59E-02	negative regulation of microtubule depolymerization
7205	9/ 30	2.60E-02	4/ 19	4.05E-02	protein kinase C activation
6810	91/ 511	2.80E-02	332/ 2903	0.00E+00	transport
165	8/ 26	3.02E-02	10/ 51	2.62E-03	MAPKKK cascade
6725	6/ 17	3.03E-02	7/ 28	2.70E-03	aromatic compound metabolism
7411	11/ 45	3.48E-02	17/ 133	4.77E-03	axon guidance
7185	7/ 22	3.49E-02	14/ 63	1.22E-06	transmembrane receptor protein tyrosine phosphatase signaling pathway
6812	36/ 187	4.41E-02	44/ 300	1.11E-07	cation transport
7214	5/ 14	4.48E-02	6/ 31	1.94E-02	gamma-aminobutyric acid signaling pathway
6468	124/ 731	5.00E-02	145/ 1176	3.38E-13	protein amino acid phosphorylation

C: human caudate-cerebellum and mouse striatum-cerebellum GO processes containing probesets with reduced RNA levels in human caudate and mouse striatum compared with human and mouse cerebellum respectively

GO ID	Human		Mouse		Function
	# sig/total probesets	p-value	# sig/total probesets	p-value	
398	97/ 170	0.00E+00	54/ 308	2.29E-07	nuclear mRNA splicing via spliceosome
6397	51/ 90	5.55E-17	80/ 424	3.01E-12	mRNA processing
8380	55/ 111	5.16E-14	5/ 17	1.61E-02	RNA splicing
6350	424/ 1530	6.16E-12	381/ 2571	0.00E+00	transcription
6406	34/ 62	2.39E-11	9/ 42	1.31E-02	mRNA export from nucleus
6355	470/ 1819	3.53E-08	472/ 3442	0.00E+00	regulation of transcription DNA-dependent
6512	102/ 306	3.74E-08	93/ 795	7.85E-03	ubiquitin cycle
6260	53/ 162	1.05E-04	41/ 214	2.54E-07	DNA replication
6281	53/ 162	1.05E-04	53/ 333	1.20E-05	DNA repair
6396	25/ 64	1.75E-04	21/ 109	1.48E-04	RNA processing
6913	11/ 22	3.95E-04	13/ 40	1.86E-07	nucleocytoplasmic transport
165	12/ 26	7.72E-04	9/ 51	4.23E-02	MAPKKK cascade
16567	76/ 266	1.00E-03	51/ 426	2.52E-02	protein ubiquitination
6310	23/ 63	1.15E-03	12/ 58	1.29E-03	DNA recombination
122	30/ 91	2.31E-03	29/ 183	9.90E-04	negative regulation of transcription from RNA polymerase II promoter
9790	11/ 27	5.63E-03	6/ 26	2.84E-02	embryonic development
46777	9/ 19	9.00E-03	10/ 57	3.47E-02	protein amino acid autophosphorylation
6916	40/ 142	1.65E-02	22/ 129	1.07E-03	anti-apoptosis
6887	13/ 39	2.81E-02	12/ 81	4.14E-02	exocytosis
6511	40/ 149	3.70E-02	32/ 257	3.73E-02	ubiquitin-dependent protein catabolism
15986	16/ 53	4.82E-02	11/ 60	7.46E-03	ATP synthesis coupled proton transport

D: human caudate-cerebellum and mouse striatum-cerebellum GO processes containing probesets with increased RNA levels in human caudate and mouse striatum compared with human and mouse cerebellum respectively

GO ID	Human		Mouse		Function
	# sig/total probesets	p-value	# sig/total probesets	p-value	
7399	131/ 410	3.45E-10	59/ 312	1.69E-12	nervous system development
7155	172/ 574	4.71E-10	103/ 828	2.81E-06	cell adhesion
7242	132/ 420	1.16E-09	128/ 834	9.77E-15	intracellular signaling cascade
7268	81/ 244	8.45E-08	21/ 123	1.50E-04	synaptic transmission
7169	43/ 122	1.04E-05	30/ 123	2.28E-11	transmembrane receptor protein tyrosine kinase signaling pathway
8152	109/ 392	3.82E-05	89/ 619	6.34E-09	metabolism
6813	55/ 173	4.20E-05	32/ 281	2.37E-02	potassium ion transport
6811	102/ 381	3.52E-04	76/ 778	4.91E-02	ion transport
30154	98/ 369	6.19E-04	74/ 624	3.38E-04	cell differentiation
16042	23/ 65	8.70E-04	14/ 99	1.48E-02	lipid catabolism
9968	21/ 58	9.22E-04	24/ 70	6.66E-16	negative regulation of signal transduction
8360	14/ 34	9.37E-04	19/ 118	8.01E-04	regulation of cell shape
6835	8/ 14	2.31E-03	7/ 23	1.82E-03	dicarboxylic acid transport
30308	16/ 44	3.09E-03	2/ 4	3.57E-02	negative regulation of cell growth
6629	75/ 286	3.51E-03	44/ 293	8.48E-06	lipid metabolism
6928	48/ 171	3.62E-03	10/ 61	2.51E-02	cell motility
42135	7/ 12	3.78E-03	3/ 8	2.22E-02	neurotransmitter catabolism
7264	61/ 232	7.11E-03	68/ 396	2.35E-11	small GTPase mediated signal transduction
6470	50/ 188	1.05E-02	51/ 278	2.44E-10	protein amino acid dephosphorylation
1516	7/ 14	1.13E-02	3/ 10	4.21E-02	prostaglandin biosynthesis
6687	6/ 11	1.13E-02	2/ 4	3.57E-02	glycosphingolipid metabolism
7218	21/ 68	1.16E-02	25/ 127	1.01E-06	neuropeptide signaling pathway
188	10/ 24	1.21E-02	8/ 34	5.16E-03	inactivation of MAPK activity
6631	25/ 85	1.39E-02	18/ 108	6.12E-04	fatty acid metabolism
7165	376/ 1715	1.45E-02	184/ 1382	5.84E-13	signal transduction
45786	39/ 144	1.52E-02	21/ 115	3.72E-05	negative regulation of progression through cell cycle
74	82/ 337	2.00E-02	51/ 407	6.07E-04	regulation of progression through cell cycle
8015	23/ 79	2.00E-02	4/ 12	1.29E-02	circulation
8154	6/ 13	2.93E-02	4/ 16	3.64E-02	actin polymerization and/or depolymerization
7411	14/ 45	2.97E-02	23/ 133	5.83E-05	axon guidance
50730	4/ 7	3.28E-02	10/ 32	1.56E-04	regulation of peptidyl-tyrosine phosphorylation
6936	33/ 126	3.81E-02	12/ 44	1.80E-06	muscle contraction
1578	2/ 2	3.96E-02	4/ 16	3.64E-02	microtubule bundle formation
7032	2/ 2	3.96E-02	4/ 15	2.91E-02	endosome organization and biogenesis
1502	6/ 14	4.29E-02	4/ 17	4.46E-02	cartilage condensation
7214	6/ 14	4.29E-02	7/ 31	1.10E-02	gamma-aminobutyric acid signaling pathway
16477	10/ 29	4.77E-02	11/ 76	2.21E-02	cell migration
8366	5/ 11	4.94E-02	8/ 17	2.42E-05	nerve ensheathment

E: human cortex-cerebellum and mouse cortex-cerebellum GO processes containing probesets with reduced RNA levels in human and mouse cortex compared with human and mouse cerebellum respectively

GO ID	Human		Mouse		Function
	# sig/total probesets	p-value	# sig/total probesets	p-value	
398	96/ 170	0.00E+00	79/ 308	0.00E+00	nuclear mRNA splicing via spliceosome
8380	55/ 111	2.00E-14	6/ 17	2.91E-03	RNA splicing
6350	429/ 1530	4.40E-14	403/ 2571	0.00E+00	transcription
6397	47/ 90	5.30E-14	106/ 424	0.00E+00	mRNA processing
6406	36/ 62	1.39E-13	8/ 42	3.37E-02	mRNA export from nucleus
6355	474/ 1819	9.34E-10	507/ 3442	0.00E+00	regulation of transcription DNA-dependent
6281	61/ 162	3.57E-08	48/ 333	3.68E-04	DNA repair
6396	29/ 64	4.90E-07	29/ 109	1.04E-10	RNA processing
6260	58/ 162	7.84E-07	61/ 214	0.00E+00	DNA replication
6376	13/ 20	2.10E-05	17/ 60	1.12E-07	mRNA splice site selection
6310	25/ 63	8.82E-05	11/ 58	4.53E-03	DNA recombination
184	11/ 19	4.03E-04	7/ 19	9.73E-04	mRNA catabolism nonsense-mediated decay
6913	12/ 22	4.62E-04	11/ 40	2.63E-05	nucleocytoplasmic transport
6357	86/ 312	1.09E-03	30/ 185	3.82E-04	regulation of transcription from RNA polymerase II promoter
122	30/ 91	1.73E-03	31/ 183	1.13E-04	negative regulation of transcription from RNA polymerase II promoter
6730	17/ 45	2.17E-03	21/ 69	3.63E-10	one-carbon compound metabolism
6270	11/ 26	3.08E-03	8/ 28	2.77E-03	DNA replication initiation
6512	82/ 306	3.47E-03	86/ 795	4.57E-02	ubiquitin cycle
8016	11/ 32	2.69E-02	7/ 27	8.98E-03	regulation of heart contraction
6605	16/ 51	2.84E-02	34/ 277	3.32E-02	protein targeting
6364	18/ 60	3.57E-02	14/ 68	4.98E-04	rRNA processing
16567	66/ 266	4.37E-02	56/ 426	1.82E-03	protein ubiquitination

F: human cortex-cerebellum and mouse striatum-cerebellum GO processes containing probesets with increased RNA levels in human and mouse cortex compared with human and mouse cerebellum respectively

GO ID	Human		Mouse		Function
	# sig/total probesets	p-value	# sig/total probesets	p-value	
7268	102/ 244	1.08E-11	21/ 123	2.83E-04	synaptic transmission
7018	37/ 71	8.31E-09	23/ 140	3.32E-04	microtubule-based movement
6813	69/ 173	2.42E-07	41/ 281	1.00E-04	potassium ion transport
7264	87/ 232	3.23E-07	80/ 396	0.00E+00	small GTPase mediated signal transduction
6886	110/ 310	4.28E-07	71/ 491	7.07E-07	intracellular protein transport
7399	139/ 410	4.48E-07	73/ 312	0.00E+00	nervous system development
6695	19/ 32	1.00E-06	17/ 47	3.93E-12	cholesterol biosynthesis
7242	139/ 420	2.31E-06	136/ 834	5.55E-17	intracellular signaling cascade
8152	131/ 392	2.38E-06	82/ 619	7.45E-06	metabolism
6811	125/ 381	1.19E-05	101/ 778	2.17E-06	ion transport
7155	171/ 574	2.43E-04	116/ 828	3.01E-09	cell adhesion
6470	64/ 188	3.96E-04	52/ 278	3.33E-10	protein amino acid dephosphorylation
6457	86/ 268	5.63E-04	53/ 397	2.05E-04	protein folding
16126	7/ 9	9.44E-04	14/ 40	7.50E-10	sterol biosynthesis
15986	22/ 53	1.12E-03	12/ 60	6.36E-04	ATP synthesis coupled proton transport
6928	57/ 171	1.44E-03	13/ 61	1.49E-04	cell motility
7165	454/ 1715	2.33E-03	202/ 1382	0.00E+00	signal transduction
7215	11/ 20	2.48E-03	3/ 8	2.44E-02	glutamate signaling pathway
7169	42/ 122	2.56E-03	25/ 123	1.03E-06	transmembrane receptor protein tyrosine kinase signaling pathway
6812	60/ 187	3.32E-03	38/ 300	4.14E-03	cation transport
7411	18/ 45	4.98E-03	25/ 133	8.47E-06	axon guidance
188	11/ 24	5.33E-03	7/ 34	2.15E-02	inactivation of MAPK activity
7548	11/ 24	5.33E-03	7/ 26	4.75E-03	sex differentiation
9968	21/ 58	1.23E-02	25/ 70	1.11E-16	negative regulation of signal transduction
6084	4/ 5	1.27E-02	4/ 10	7.03E-03	acetyl-CoA metabolism
42552	4/ 5	1.27E-02	8/ 27	1.32E-03	myelination
17157	3/ 3	1.33E-02	7/ 18	4.19E-04	regulation of exocytosis
30517	3/ 3	1.33E-02	4/ 14	2.55E-02	negative regulation of axon extension
8610	6/ 10	1.51E-02	27/ 137	1.02E-06	lipid biosynthesis
15992	29/ 87	1.69E-02	22/ 93	6.36E-08	proton transport
6468	197/ 731	1.73E-02	174/ 1176	9.99E-16	protein amino acid phosphorylation
7266	12/ 30	1.77E-02	11/ 67	9.39E-03	Rho protein signal transduction
30866	7/ 13	1.81E-02	9/ 32	1.01E-03	cortical actin cytoskeleton organization and biogenesis
7218	23/ 68	2.44E-02	29/ 127	2.57E-09	neuropeptide signaling pathway
45786	44/ 144	2.58E-02	19/ 115	9.02E-04	negative regulation of progression through cell cycle
6936	39/ 126	2.71E-02	10/ 44	3.12E-03	muscle contraction
6835	7/ 14	2.89E-02	7/ 23	2.22E-03	dicarboxylic acid transport
6887	14/ 39	3.63E-02	14/ 81	2.09E-03	exocytosis
6182	6/ 12	4.26E-02	4/ 12	1.44E-02	cGMP biosynthesis
30154	101/ 369	4.64E-02	83/ 624	5.49E-06	cell differentiation
